# Causal relationships of neonatal jaundice, direct bilirubin and indirect bilirubin with autism spectrum disorder: A two-sample Mendelian randomization analysis

**DOI:** 10.3389/fpubh.2023.1137383

**Published:** 2023-04-13

**Authors:** Li-wen Chen, Yi Zhang, Dou-dou Xu, Yang Wang, Hui Gao

**Affiliations:** ^1^Department of Pediatrics, The First Affiliated Hospital of Anhui Medical University, Hefei, Anhui, China; ^2^Key Laboratory of Population Health Across Life Cycle, Ministry of Education of the People's Republic of China, Anhui Medical University, Hefei, Anhui, China; ^3^Ministry of Education Key Laboratory for Full Life Cycle Population Health, Anhui Medical University, Hefei, Anhui, China

**Keywords:** autism spectrum disorder, neonatal jaundice, indirect bilirubin (IBIL), direct bilirubin, Mendelian randomization (MR)

## Abstract

**Background:**

Multiple systematic reviews and meta-analyses have examined the association between neonatal jaundice and autism spectrum disorder (ASD) risk, but their results have been inconsistent. This may be because the included observational studies could not adjust for all potential confounders. Mendelian randomization study can overcome this drawback and explore the causal relationship between the both.

**Methods:**

We used the data of neonatal jaundice, direct bilirubin (DBIL), indirect bilirubin (IBIL), and ASD collected by genome-wide association study (GWAS) to evaluate the effects of neonatal jaundice, DBIL and IBIL on ASD by using a two-sample Mendelian randomized (MR). The inverse variance-weighted method (IVW) was the main method of MR analysis in this study. Weighted median method, MR-Egger regression and mendelian randomization pleiotropy residual sum and outlier (MR-PRESSO) test were used for sensitivity analysis.

**Results:**

There was no evidence of an effect of neonatal jaundice (OR, 1.002, 95% CI, 0.977–1.027), DBIL (OR, 0.970, 95% CI, 0.884–1.064) and IBIL (OR, 1.074, 95% CI, 0.882–1.308) on ASD risk by IVW test. In the weighted median method, MR-Egger regression and leave-one-out analysis, the results were robust and no heterogeneity or pleiotropy was observed.

**Conclusions:**

We found that neonatal jaundice, DBIL and IBIL were not associated with ASD in this study. However, this paper did not explore the effect of severity and duration of jaundice on ASD in different ethnic populations, which may require further research.

## 1. Introduction

Autism spectrum disorder (ASD) is a heterogeneous group of neurodevelopmental conditions, characterized by difficulties in social communication and interaction, as well as abnormally limited, repetitive behaviors and interests (1). In recent years, the prevalence of ASD has increased significantly. The prevalence of ASD in children aged 3–17 years in the United States in 2016 was 2.76%. By 2020, the prevalence of ASD had increased to 3.49% (2, 3). A nationwide multicenter population-based study (142,086) conducted in China during 2014–2016, and an estimated prevalence of 0.70% has been reported for children aged 6–12 years (4). The increasing prevalence of ASD is placing enormous pressure on health and social services, families and education (5, 6). Currently, the etiology of ASD is not clear, but it is believed that genetic factors, environmental factors (such as mercury, radiation, diesel waste, etc.), changes in neural connections, perinatal factors (such as age of parents, maternal medication, infection, etc.) and postpartum factors (such as meningitis, low birth weight, etc.) are associated with the pathogenesis of ASD (6, 7).

Neonatal jaundice is a common disease in the neonatal period. Jaundice occurs in about 60% of term infants and 80% of preterm infants (8). Bilirubin is produced from heme and, to a certain extent, is neuroprotective because of its free radical scavenging effect. Unconjugated bilirubin is a fat-soluble molecule that easily crosses the blood-brain barrier, and when it reaches a high level, it may cause a range of neurological symptoms (9, 10). Bilirubin deposition may cause neuropathological changes in the cerebellum and hippocampus (11–13), which have also been found in ASD patients (14, 15). Therefore, it is possible that there is an association between neonatal jaundice and ASD (16).

Jenabi et al., in a systematic review including 21 studies, reported that neonatal jaundice was associated with ASD in children (odds ratio (OR), 1.35, 95% confidence interval (CI), 1.02–1.68, risk ratio (RR), 1.39, 95% CI, 1.05–1.74) (17). However, Kujabi et al., based on the quality assessment of the literature, did not support the association between neonatal jaundice and ASD in 6 studies with low risk of bias (RR, 1.09, 95% CI, 0.99–1.20, OR, 1.29, 95% CI, 0.95–1.76) (18). In a systematic review including 13 observational studies, Amin et al. reported that neonatal jaundice diagnosed based on serum total bilirubin concentration was associated with an increased risk of ASD (OR, 1.43, 95% CI 1.22–1.67), but unconjugated bilirubin may be a better predictor of neurotoxicity in preterm infants than total bilirubin (16). These system reviews were mainly analyzed on the basis of observational studies, so it is difficult to avoid the interference of confounding factors. For example, hospitalized newborns are more likely to be diagnosed with jaundice, whereas non-hospitalized newborns are at much lower risk (18). Therefore, investigating the causal association of neonatal jaundice with ASD should adjust for all covariates that influence neonatal hospitalization and ASD occurrence. Including woman's first child, parental age, neonatal comorbidity, complicated delivery, child's sex, Apgar score, gestational age, birth weight and year, etc. (18). Most clinical epidemiological research methods, such as case-control studies and cohort studies, have difficulty in controlling all potential confounding factors (19), while mendelian randomization (MR) studies can solve this problem.

MR is a method that uses genetic variants as instrumental variables to assess causal relationships between exposure or risk factors and clinical outcomes of interest (20). MR is similar to randomized controlled trials (RCTs) because segregation of genetic variants is randomly assigned, and is independent of environmental factors. Thus, known and unknown confounders were equally distributed across the groups (21). Moreover, the data of MR study can be derived from Genome-wide association studies (GWAS). Therefore, MR study has the advantages of convenience, rapidity, large sample size, avoiding confounding to the greatest extent and avoiding reverse causality (22, 23). In this study, we investigated the causal effects of neonatal jaundice, direct bilirubin (DBIL) and indirect bilirubin (IBIL) on ASD by using a two-sample MR.

## 2. Methods

### 2.1. Study design and data sources

In this study, two-sample MR was used to evaluate the causal relationship between neonatal jaundice, DBIL, IBIL and the risk of ASD. Effective instrument variables [in this study are single nucleotide polymorphisins (SNPs)] need to satisfy three core assumptions: (i) assumption of relevance: SNPs are correlated with exposure, (ii) assumption of independence: SNPs are not related to confounders, (iii) assumption of exclusivity: SNPs are related to outcomes only by exposure (24).

GWAS is a research method to study the correlation between genetic mutations and phenotypes (25). The GWAS databases aggregate genotype and phenotype associations from genome-wide association studies. We obtained the latest and largest sample size GWAS summary data for exposures from IEU OpenGWAS project (mrcieu.ac.uk). The exposures data's that we acquired included neonatal jaundice (133 cases and 218,608 controls), DBIL (6,961 cases and 6,961 controls) and IBIL (6,972 cases and 6,972 controls). The study population for neonatal jaundice data was European, while the study population for DBIL and IBIL was the South Asian. Symptoms of jaundice can be observed in neonates with total serum bilirubin levels above 5.0 mg/dl (26). However, the bilirubin threshold for intervention is affected by factors such as gestational age, postnatal age, ABO/Rh hemolytic disease, and glucose-6-phosphate dehydrogenase (G6PD) deficiency (27). In general, the bilirubin threshold requiring clinical intervention was reached when the total serum bilirubin level in term infants exceeded 5 mg/dl on the 1st day after birth, 10 mg/dl on the 2nd day, or 13 mg/dl on the third day and beyond (28). Hepatobiliary injury is indicated when direct bilirubin levels exceed 1.0 mg/dL, or represent more than 20% of total bilirubin (26). GWAS summary data for result (in this study is ASD) were also obtained from IEU OpenGWAS project (mrcieu.ac.uk). The GWAS data of ASD included 4,949 experimental and 5,314 control subjects, with a European population.

### 2.2. Instrumental variable selection criteria

SNPs were initially selected from relevant GWAS databases using the following criteria: (i) *p*-value of <5 × 10^−8^, ii) a linkage disequilibrium (LD) r^2^ of <0.001, within a 10,000 kb window. According to this standard, 3 SNPs of DBIL and 4 SNPs of IBIL were screened out, and no SNPs meeting the standard for neonatal jaundice were found. For better study, we relaxed the *p*-value of the SNP associated with neonatal jaundice to 5 × 10^−6^, and adjusted the *p*-value of the SNP associated with DBIL and IBIL to 5 × 10^−7^. Previous studies have used similar thresholds for instrumental variables (29, 30). F statistic can be used to assess the instrument strength for each of the exposed SNPs, which was calculated by the formula β^2^/σ^2^ (β indicates the association between SNP and exposure) (29). Typically, F statistic >10 is considered to be strongly associated with exposure (31).

Afterwards, the information of SNPs which were selected through the above process in the outcome was extracted and discarded if it were not available in the outcome. A SNP (rs6750992) of IBIL lacked σ (means variance) and was abandoned when MR analysis was performed. Next, we combined the exposure and outcome data which were used for the MR analysis.

### 2.3. MR analysis

The inverse variance-weighted method (IVW) was the main method of MR analysis in this study, and its basic assumption is that all selected instrumental variables are valid, that is, the three core assumptions of MR need to be satisfied. If the SNPs have horizontal pleiotropy, they violate the exclusivity assumption and may cause a large bias in the IVW results (32).

Weighted median method and MR-Egger regression were used to carry out sensitivity analysis. The weighted median method is to make estimates of causal effects under the assumption that less than 50% of the SNPs are null (33). MR-Egger regression can also give consistent causal effect estimates when all SNPS are considered as invalid instrumental variables. And it can be used to test for pleiotropy bias (34).

Mendelian randomization pleiotropy residual sum and outlier (MR-PRESSO) test was also a way to assess sensitivity. MR-PRESSO can identify outliers for horizontal pleiotropy in MR summary data, remove outliers to correct horizontal pleiotropy, and compare the differences between the MR analysis results before and after correction (35). In this study, we also conducted the simple mode, weighted mode, Cochrane's Q statistic and leave-one-out analysis. All the above analyses were performed in Rstudio software using the R packages “TwosampleMR” and “MR-PRESSO”.

### 2.4. Statistical power

We calculated the statistical power on https://shiny.cnsgenomics.com/mRnd/ (36). The power calculation required the total value of the R^2^ (the proportion of variance explaining the association between the SNP and the exposure). We calculate it by the following formula: 2^*^EAF^*^(1-EAF)^*^β^2^ (37).

## 3. Results

### 3.1. Overview

After selection, 8 SNPs of neonatal jaundice, 5 SNPs of DBIL and 6 SNPs of IBIL could be used for MR analysis. In this study, the F statistics of the selected SNPs were all >10. Final analysis data are shown in Table 1.

**Table 1:** Causal effects of neonatal jaundice, DBIL, and IBIL on ASD in Mendelian randomization analysis.

**Outcome**	**Exposure**	**SNP**	**Chr**.	**Effect allele**	**Reference allele**	**Exposure**			**Outcome**			**F statistics**
						**Beta**	**SE**	***P***	**Beta**	**SE**	***P***	
ASD	Neonatal jaundice	rs11697126	20	C	G	1.883	0.382	8.39E-07	0.004	0.052	0.943	24.27
ASD	Neonatal jaundice	rs139014478	6	T	G	4.848	1.053	4.18E-06	0.054	0.173	0.754	21.182
ASD	Neonatal jaundice	rs192582383	4	C	T	4.012	0.836	1.61E-06	0.091	0.142	0.519	23.006
ASD	Neonatal jaundice	rs2304838	17	T	C	−0.678	0.146	3.62E-06	0.017	0.031	0.582	21.451
ASD	Neonatal jaundice	rs2991346	1	A	G	−0.603	0.131	4.21E-06	0.022	0.028	0.420	21.156
ASD	Neonatal jaundice	rs62543126	9	C	T	3.556	0.778	4.81E-06	0.11	0.101	0.276	20.91
ASD	Neonatal jaundice	rs75636355	20	C	T	1.924	0.414	3.43E-06	−0.002	0.092	0.987	21.561
ASD	Neonatal jaundice	rs9310503	3	T	G	0.632	0.132	1.61E-06	−0.033	0.028	0.237	23.001
ASD	DBIL	rs1124251	12	A	G	0.135	0.024	1.12E-08	−0.015	0.031	0.618	32.619
ASD	DBIL	rs12982373	19	T	C	0.264	0.051	1.88E-07	0.014	0.070	0.836	27.158
ASD	DBIL	rs34847903	2	G	A	0.089	0.017	1.41E-07	−0.010	0.034	0.763	27.695
ASD	DBIL	rs6709005	2	C	T	0.105	0.017	1.13E-09	0.030	0.037	0.436	37.109
ASD	DBIL	rs887829	2	T	C	0.59	0.017	1.00E-200	−0.020	0.030	0.489	1,238.822
ASD	IBIL	rs11563216	2	G	C	0.14	0.018	2.98E-14	−0.012	0.040	0.764	57.724
ASD	IBIL	rs116149023	2	T	C	−0.194	0.023	7.11E-18	−0.043	0.040	0.276	74.145
ASD	IBIL	rs12982373	19	T	C	0.281	0.054	1.74E-07	0.014	0.070	0.836	27.303
ASD	IBIL	rs61745029	5	T	C	0.266	0.052	3.68E-07	−0.039	0.060	0.509	25.858
ASD	IBIL	rs7132068	12	C	G	−0.123	0.024	3.74E-07	−0.020	0.031	0.509	25.835
ASD	IBIL	rs931602	4	A	G	0.096	0.018	1.49E-07	0.022	0.030	0.471	27.599

ASD, autism spectrum disorder; DBIL, direct bilirubin; IBIL, indirect bilirubin. The *p*-value of the SNPs for neonatal jaundice was 5E-06 and the *p-*value of the SNPs for DBIL and IBIL was 5E-07.

### 3.2. Casual estimates of neonatal jaundice, DBIL and IBIL on ASD

When *p*-value < 5 × 10^−6^, no significant association was found between neonatal jaundice and ASD by IVW test (OR, 1.002, 95% CI, 0.977–1.027, *p* = 0.885). Similarly, no significant relationship was found between DBIL and ASD (OR, 0.970, 95% CI, 0.884–1.064, *p* = 0.514) when *p*-value < 5 × 10^−7^, and the relationship between IBIL and ASD [OR = 1.074, 95% CI = (0.882, 1.308), *p* = 0.475] was the same result. The above results are presented in Table 2.

**Table 2:** Casual estimates of neonatal jaundice, DBIL and IBIL on ASD.

**Method**	**Number of IVs**	**MR analysis**		**Pleiotropy**		**Heterogeneity**	
		**OR (95% CI)**	***P***	**MR-egger intercept**	***P***	**Cochran's Q**	***P***
**Neonatal jaundice on ASD**							
IVW	8	1.002 (0.977, 1.027)	0.885			4.039	0.775
MR-Egger	8	1.032 (0.992, 1.074)	0.173	−0.045	0.109	0.513	0.998
Weighted median	8	1.006 (0.971, 1.042)	0.745				
Simple mode	8	1.009 (0.960, 1.061)	0.728				
Weighted mode	8	1.013 (0.971, 1.057)	0.58				
**DBIL on ASD**							
IVW	5	0.970 (0.884, 1.064)	0.514			1.041	0.904
MR-egger	5	0.960 (0.831, 1.109)	0.619	0.004	0.874	1.011	0.799
Weighted median	5	0.965 (0.880, 1.059)	0.453				
Simple mode	5	0.927 (0.747, 1.150)	0.527				
Weighted mode	5	0.964 (0.875, 1.063)	0.507				
**IBIL on ASD**							
IVW	6	1.074 (0.882, 1.308)	0.475			2.201	0.821
MR-Egger	6	0.896 (0.516, 1.555)	0.715	0.031	0.527	1.722	0.787
Weighted median	6	1.120 (0.876, 1.430)	0.366				
Simple mode	6	1.196 (0.831, 1.723)	0.379				
Weighted mode	6	1.199 (0.840, 1.710)	0.363				

ASD, autism spectrum disorder; DBIL, direct bilirubin; IBIL, indirect bilirubin. The *p*-value of the SNPs for neonatal jaundice was 5E-06 and the *p*-value of the SNPs for DBIL and IBIL was 5E-07. Results were obtained from a two-sample Mendelian analysis. Outliers were tested by MR-PRESSO.

### 3.3. Sensitive, heterogeneity and pleiotropy analysis

MR-PRESSO and MR-Egger intercept were used to test horizontal pleiotropy. If abnormal SNPs are found by MR-PRESSO method, they need to be eliminated and re-analyzed by MR. No abnormal SNPs were identified in this study. The results of MR-Egger intercept are shown in Table 2. All *p*-values were >0.05, therefore, there was no horizontal pleiotropy in the causal estimates of neonatal jaundice, DBIL and IBIL on ASD. Heterogeneity was tested using the Cochran's Q test. The *p*-value of Q based on IVW method and MR-Egger method is >0.05 in this study (the specific results are shown in Table 2), which indicates that there is no heterogeneity in the above three causal studies.

**Figure 1:**
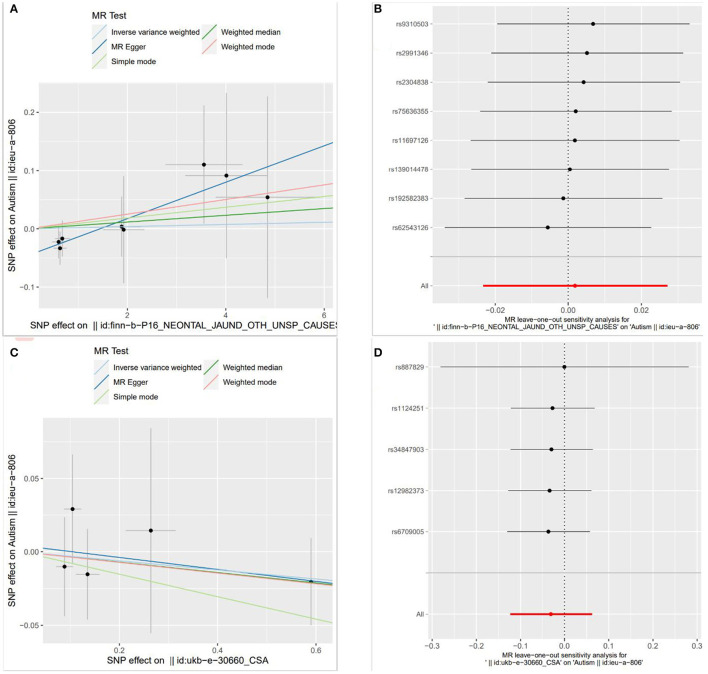
Scatter plots and leave-one-out plot of the causal estimates of neonatal jaundice and direct bilirubin (DBIL) on autism spectrum disorder (ASD) [Scatter plots of MR analysis were calculated by IVW, MR-egger, weighted median, single mode and weighted mode methods. The slopes indicate the causality of each method. **(A)** Illustrates the relationship between neonatal jaundice and ASD, and **(C)** illustrates the relationship between DBIL and ASD; Leave-one-out analyses demonstrated the robustness of the associations of neonatal jaundice **(B)** and DBIL **(D)** with ASD].

**Figure 2:**
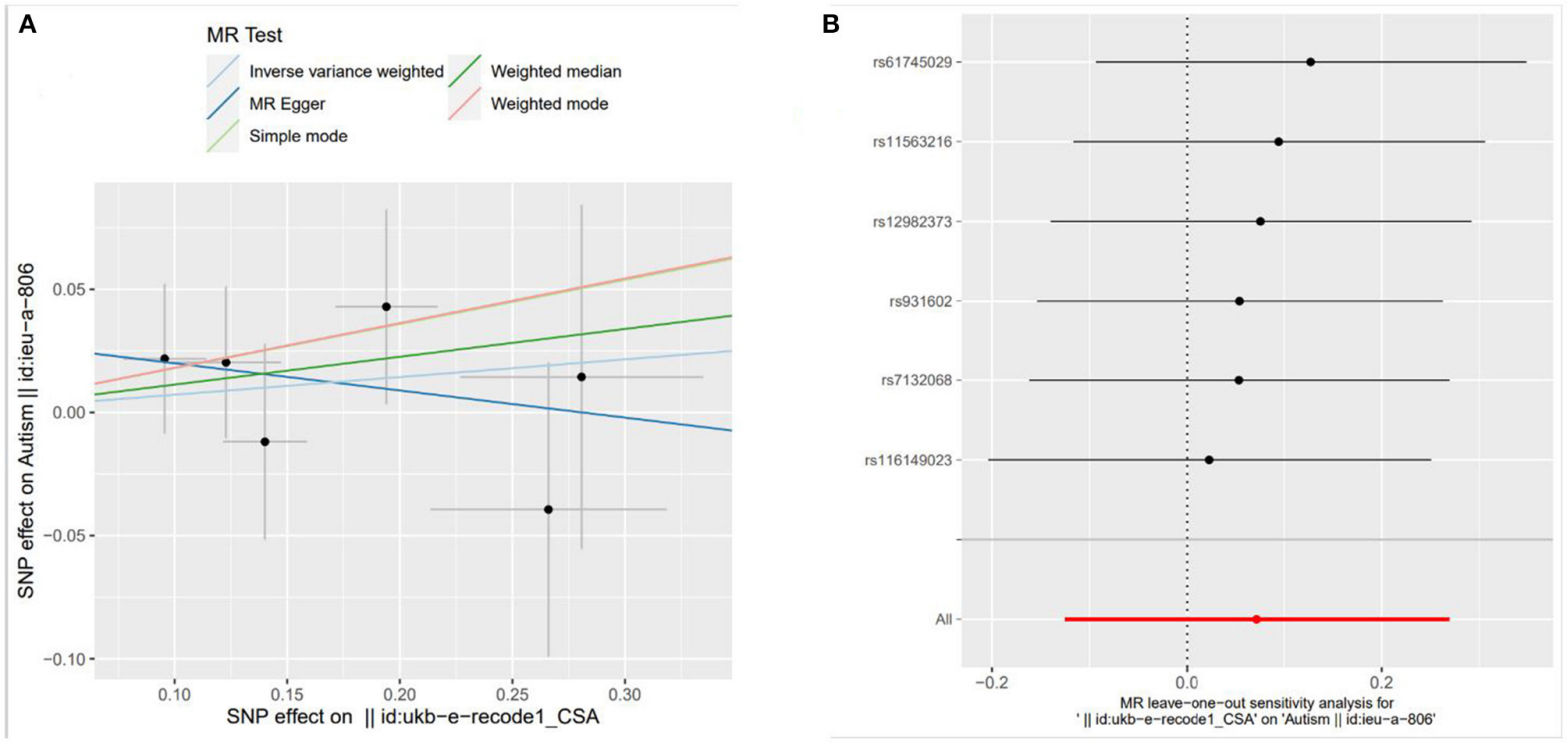
Scatter plots and leave-one-out plot of the causal estimates of indirect bilirubin (IBIL) on autism spectrum disorder [Scatter plots of MR analysis were calculated by IVW, MR-egger, weighted median, single mode and weighted mode methods. The slopes indicate the causality of each method. **(A)** Illustrates the relationship between IBIL and ASD; Leave-one-out analyses demonstrated the robustness of the associations of IBIL **(B)** with ASD].

The weighted median method and MR-Egger regression were used to test the sensitivity in the paper, and the results were consistent with the results of IVW. The results obtained by simple mode and weighted mode methods were also consistent with those of the above three methods. This illustrates the robustness of the results of this study. The robustness was also verified by leave-one-out analysis. Scatter plots of the causal estimates of neonatal jaundice, DBIL and IBIL on ASD and the leave-one-out plot are shown in Figures 1, 2.

### 3.4. Statistical power

The statistical power of MR analysis for the causal effect of neonatal jaundice, DBIL and IBIL on ASD was 0.05, 0.10, and 0.49, respectively.

## 4. Discussion

We investigated the causal association of neonatal jaundice, DBIL and IBIL with ASD using a mendelian randomization study approach. We found genetic proof that neonatal jaundice, DBIL and IBIL had no significant causal relationship with ASD.

Neonatal jaundice is usually a mild disease (38) when bilirubin level is in a safe range. Bilirubin increases in newborns, especially during the 1st week of life, due to the immature liver enzyme system, increased red blood cell breakdown and the presence of enterohepatic circulation. Neurotoxic effects that occur when bilirubin levels are above age-appropriate thresholds may lead to kernicterus and may manifest clinically in changes in muscle tone, abnormal hearing, and abnormalities in speech and language (39, 40). Kernicterus often affects the globus pallidus, but can also involve the subthalamic nucleus, brain stem, hippocampus and cerebellum. The resulting pathological changes in the brain include cerebellar damage (reduced number of Purkinje cells) and impaired pathways between cerebellar and cortical neurons, which is similar to the pathogenesis of ASD (7, 39). High values of bilirubin, especially extremely high values of bilirubin, may increase the worry of family members, and if neonatal bilirubin is related to ASD, this may increase the worry and burden of family members. The results of this paper may provide some reassurance to them.

Result of this paper is consistent with the results of the literature review by Kujabi et al., who rigorously evaluated the included articles (18). However, it is inconsistent with the results of the literature review by Amin et al. and Jenabi et al. (16, 17). But many of the included articles did not control for confounding factors, some articles did not clearly define jaundice and some information on bilirubin levels was retrieved from parents, which may have led to various biases (16, 17). In this article, we only considered the presence or absence of jaundice and do not account for the severity or duration of jaundice or geographic differences in race (41), which may have contributed to our lack of positive findings. Amin et al. and Jenabi et al. found a link between increased severity of jaundice and a possible increased risk of ASD (16, 17), suggesting that more severe jaundice is associated with a greater likelihood of developing ASD. Without stratification of jaundice by severity, including mild jaundice cases in the analysis may have attenuated the effect estimates between jaundice and ASD. In the course of the study, we did not consider this point. The duration of jaundice, which can worsen over time in the absence of intervention, is also an important factor, and this may increase ASD risk (41). Ethnic and geographic differences are also important factors. The prevalence of neonatal jaundice and kernicterus was different in different nationalities and regions. For example, black infants have a higher prevalence of kernicterus (42). Other groups, such as persons of African, Asian, Mediterranean, or Middle Eastern ancestry, may have an increased risk of neonatal hyperbilirubinemia because of G6PD deficiency (43). A higher threshold of concern for the evaluation of jaundice in such neonates may lead to an increased incidence of neurologic impairment. And infants in low-income and middle-income countries are more likely to develop severe jaundice due to lower levels of diagnosis, lower levels of care, delayed or inappropriate treatment (39, 44). However, differences in study populations may increase or decrease the effect estimates between neonatal jaundice and ASD (41). The GWAS databases we used were only European or South Asian, which may not be so representative. In addition, jaundice is highly prevalent in the neonatal period, and the GWAS database for neonatal jaundice used in our study had only 133 cases compared with 218,606 controls. Therefore, there may be an information deficit in the GWAS data we used, which may underestimate the effect of neonatal jaundice on ASD.

There are some limitations of this study. First, because the original screening criteria of *p* < 5 × 10^−8^ were set, which reduced the number of SNPs available for analysis, we relaxed the screening criteria, which may have introduced instrumental bias. Although the F-statistics showed that the SNPs in the final analysis were all strong instrumental variables. Second, the study populations of GWAS data for DBIL and IBIL are not consistent with those of GWAS data for ASD, which may lead to population heterogeneity bias. Moreover, the sample size of GWAS data on neonatal jaundice is relatively small. Third, although evaluation by the MR-Egger intercept, the MR-PRESSO method, and leave-one-out analysis did not reveal significant pleiotropy, this possibility cannot be excluded. Fourth, the statistical power of our study was low and did not reach the target power (0.80). This may be related to the insufficient sample size, so a more in-depth study is needed. In addition, the duration and severity of jaundice and the correlation with age of onset, severity of autism, and predictive parameters were not taken into account in this study, which also requires further investigation. Finally, the GWAS data we used is relatively limited in population, and the generalization of the results to other populations may require larger and more comprehensive studies.

## 5. Conclusion

In conclusion, in this study, we did not find a causal relationship between neonatal jaundice and ASD, nor did DBIL and IBIL. Data from a larger sample may be needed to verify the causal relationship between neonatal jaundice and ASD. Further exploration of the impact of severity and duration of jaundice on ASD in different ethnic populations is also needed.

## Data availability statement

The datasets presented in this study can be found in online repositories. The names of the repository/repositories and accession number(s) can be found in the article/supplementary material.

## Author contributions

L-wC participated in conceptualizing and writing the draft. YZ and D-dX were responsible for methodology and statistical analysis. YW participated in writing review and editing. While HG was mainly responsible for funding acquisition, writing review, and editing. All authors agreed to the final version as submitted.
